# Genetic susceptibility to hereditary non-medullary thyroid cancer

**DOI:** 10.1186/s13053-022-00215-3

**Published:** 2022-03-07

**Authors:** Tina Kamani, Parsa Charkhchi, Afshan Zahedi, Mohammad R. Akbari

**Affiliations:** 1 Women’s College Research Institute, University of Toronto, 76 Grenville St. Room 6421, Toronto, ON M5S 1B2 Canada; 2 Institute of Medical Science, Faculty of Medicine, University of Toronto, Toronto, ON M5S 1A8 Canada; 3 Dalla Lana School of Public Health, University of Toronto, Toronto, ON M5T 3M7 Canada

**Keywords:** Thyroid cancer, Familial non-medullary thyroid cancer, Germline mutations, *FOXE1*, *HABP2*, *SRGAP1*, *DIRC3*, *NRG1*

## Abstract

Non-medullary thyroid cancer (NMTC) is the most common type of thyroid cancer. With the increasing incidence of NMTC in recent years, the familial form of the disease has also become more common than previously reported, accounting for 5–15% of NMTC cases. Familial NMTC is further classified as non-syndromic and the less common syndromic FNMTC. Although syndromic NMTC has well-known genetic risk factors, the gene(s) responsible for the vast majority of non-syndromic FNMTC cases are yet to be identified. To date, several candidate genes have been identified as susceptibility genes in hereditary NMTC. This review summarizes genetic predisposition to non-medullary thyroid cancer and expands on the role of genetic variants in thyroid cancer tumorigenesis and the level of penetrance of NMTC-susceptibility genes.

## Introduction

**Fig. 1:**
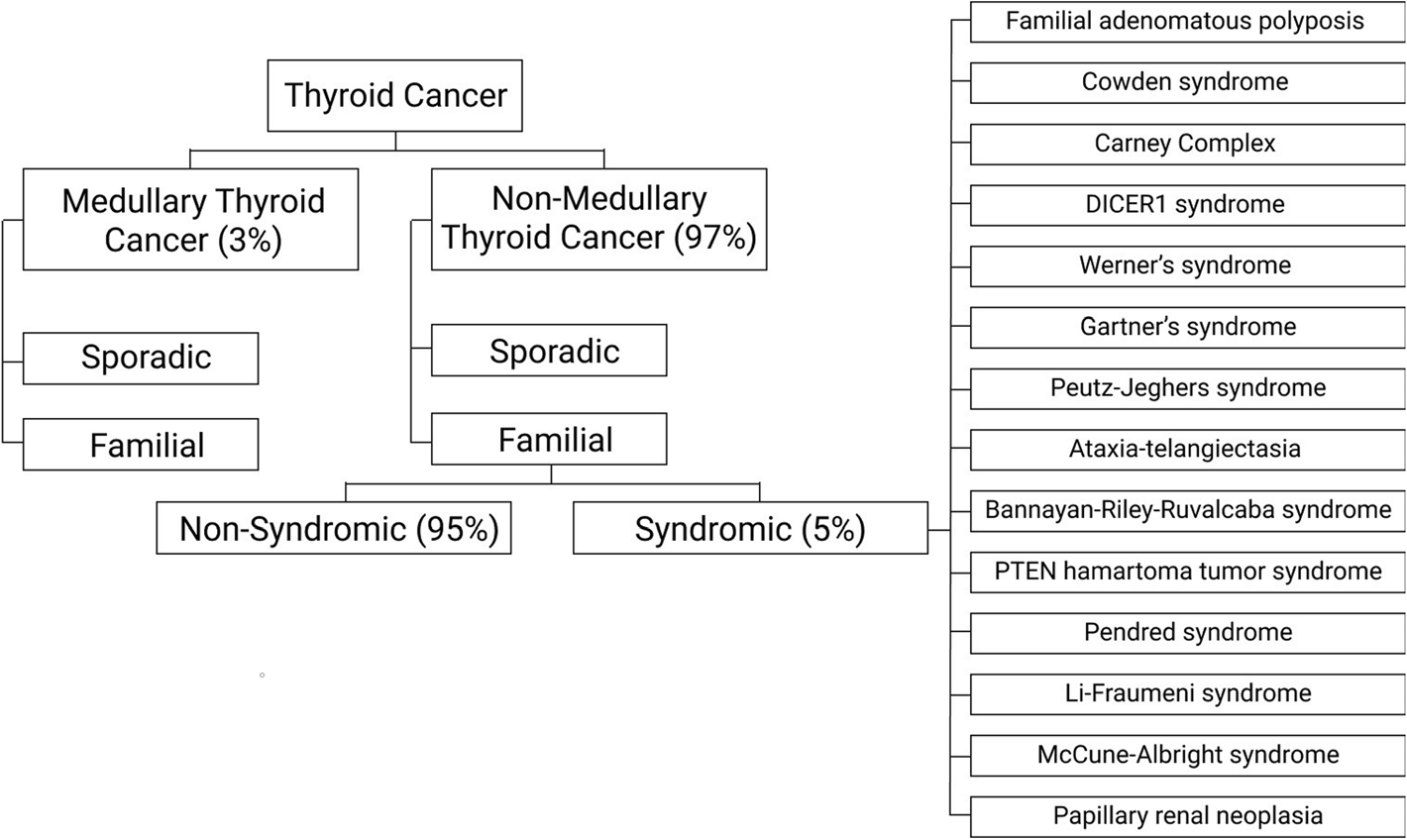
Overview of thyroid cancer subtype classification

Thyroid cancer is the most common endocrine malignancy [1], with its global incidence rate increasing substantially in the past four decades [2]. Thyroid cancers can originate due to the accumulation of genetic mutations in para-follicular or follicular cells. Thyroid cancers originating from para-follicular calcitonin-producing C cells are known as medullary thyroid carcinoma (MTC) and account for 5% of all cases, whereas the more common type of thyroid cancer arises from follicular cells and is known as non-medullary thyroid cancer [1]. The majority of NMTC are differentiated thyroid cancers (DTC) which include papillary and follicular thyroid cancers. Papillary thyroid cancer (PTC) accounts for more than 85% of NMTC cases and follicular thyroid cancer (FTC) accounts for 10–15% of NMTC cases. The rare forms of NMTC are poorly differentiated thyroid carcinomas and anaplastic thyroid carcinomas [3, 4]. Over 90% of all thyroid cancers are sporadic and arise from somatic genetic changes [5]. The remaining are familial forms of NMTC and MTC. Familial MTC (FMTC) has well-known genetic alterations and genotype-phenotype correlations. On the contrary, the genetic causes of familial NMTC (FNMTC), or familial follicular cell-derived carcinoma are poorly understood [6, 7]. FNMTC is clinically defined as the presence of the disease in two or more first-degree relatives of the patient. FNMTC can further be classified as syndromic or non-syndromic, depending on whether the thyroid cancer is the primary cancer (non-syndromic) or as a part of one of many constellations of tumours in kindreds (syndromic FNMTC) [8]. Hereditary cancer syndromes associated with FNMTC account for 5% of all familial cases and include Familial adenomatous polyposis, Cowden syndrome, Carney complex, Werner syndrome, DICER1 syndrome, Ataxia-telangiectasia, Bannayan-Riley-Ruvalcaba syndrome, Li-Fraumeni syndrome, Peutz-Jeghers syndrome, and Pendred syndrome  (Fig. 1) [9].

According to the Swedish family cancer database, the proportion of cancer susceptibility accounted for by genetic factors was the highest for thyroid cancer among 15 cancer sites [10]. Additionally, family and twin studies from Utah and Sweden suggested thyroid cancer as one of the most heritable cancers displaying Mendelian inheritance, with reported risks of 8- to 12-fold higher for first-degree relatives of thyroid cancer patients compared to the general population [11–13] . A family cohort of the Norwegian cancer registry database estimated that the familial risk ratio of NMTC in affected first-degree relatives is 5.2 for men and 4.9 for women [14, 15]. Similarly, Lin et al. identified the family structures of 38,686 NMTC patients in Taiwan. The prevalence of NMTC in the general population and in first-degree relatives of NMTC patients were 0.16% and 0.64%, respectively. This shows a 5.47-fold increased risk for NMTC for first-degree family members [16]. FNMTC patients present with more aggressive disease at a younger age compared to sporadic cases, this includes larger tumours with more lymph node involvement [17–19]. However, studies have shown no significant increase in risk of recurrence or disease-related mortality in FNMTC cases compared to sporadic cases [20–22]. As a result, the evidence for a worst disease outcome in FNMTC compared to sporadic cases is conflicting [18, 23]. Additionally, the second generation of FNMTC patients present at a younger age with more severe symptoms, indicating the presence of clinical anticipation [24]. In a prospective cohort study, at-risk relatives of twenty-five kindreds with two or more members affected by FNMTC were screened with neck ultrasound and fine-needle aspiration biopsy of thyroid nodules. The results indicated the presence of thyroid cancer in 4.6% of families with two affected members and 22.7% of families with three or more affected members. The tumours that were identified with screening were smaller in size, had less lymph node metastases, and required less extensive treatments. Therefore, the early detection of FNMTC can potentially improve the treatment outcome [25].

Despite the solid evidence for the heritability of thyroid cancer, only a handful of variants have been convincingly associated with a higher risk of this cancer. The high heritability of thyroid cancer is likely due to the contributions of rare but high-penetrance mutations in some cases or common but low-penetrance variants in others [26]. The present study aimed to summarize the literature regarding variants associated with higher risk of hereditary NMTC and provide a more extensive background on the penetrance, molecular function and functional consequences of these mutations, which can further clarify the etiology of thyroid cancer and aid in the identification of disease risk in family members of NMTC patients.

## Genetic variants associated with risk of non-medullary thyroid cancer

Approximately 5–15% of NMTC cases occur due to germline mutations [17]. Genetic variants conferring risk of complex disorders such as cancer are either rare mutations with moderate to high penetrance or common variants with low penetrance. The genetic predisposition to NMTC seems to be relatively strong based on the previous case-control studies. To date, multiple susceptibility genes have been identified through genome-wide association studies (GWAS) (Table 1). Methods such as family-based exome sequencing, next generation sequencing and linkage studies also identified several susceptibility loci associated with NMTC. Genetic variants from these genes have been classified based on their level of penetrance using odds ratio (OR) compiled from previous case-control studies. In this review, variants with an OR lower than 1.5 were classified as low penetrance mutations, and those with an OR between 1.5 and 2.5 were classified as moderate penetrance and highly penetrant mutations were categorized as variants with an OR greater than 2.5 [34].

**Table 1:** Variants associated with increased risk for non-medullary thyroid cancer in various populations identified by GWAS

Genes	Chr.	Reference	variant	Population	Type of tumour^**a**^	OR	***P*** value
MBIP	14q13.3	Gudmundsson et al. (2012) [27]	rs116909374	Iceland	NMTC	2.09	4.6 × 10^−11^
		Gudmundsson et al. (2017) [28]	rs116909374	European ancestry	NMTC	1.81	1.1 × 10^− 16^
		Son et al. (2017) [29]	rs34081947	Korean	PTC	1.28	2.4 × 10^− 4^
		Son et al. (2017) [29]	rs944289	Korean	PTC	1.24	1.41 × 10^− 3^
PCNLX2	1q42.2	Gudmundsson et al. (2017) [28]	rs12129938	Iceland, Spain, United States, Netherlands	NMTC	1.32	4.0 × 10^− 11^
LRRC34, TERC	3q26.2	Gudmundsson et al. (2017) [28]	rs6793295	Iceland, Spain, United States, Netherlands	NMTC	1.23	2.7 × 10^− 8^
TERT	5p15.33	Gudmundsson et al. (2017) [28]	rs10069690	Iceland, Spain, United States, Netherlands	NMTC	1.20	3.2 × 10^− 7^
EPB41L4A, NREP	5q22.1	Gudmundsson et al. (2017) [28]	rs73227498	Iceland, Spain, United States, Netherlands	NMTC	1.37	3.0 × 10^− 10^
OBFC1	10q24.33	Gudmundsson et al. (2017) [28]	rs7902587	Iceland, Spain, United States, Netherlands	NMTC	1.41	5.4 × 10^− 11^
SMAD3	15q22.33	Gudmundsson et al. (2017) [28]	rs2289261	Iceland, Spain, United States, Netherlands	NMTC	1.23	3.1 × 10^− 9^
IMMP2L	7q31.1	Köhler et al. (2013) [30]	rs10238549	Italy	DTC	1.27	4.1 × 10^− 6^
		Köhler et al. (2013) [30]	rs7800391	Italy	DTC	1.25	5.7 × 10^− 6^
RARRES1	3q25.32	Köhler et al. (2013) [30]	rs7617304	Italy	DTC	1.25	4.6 × 10^− 5^
SNAPC4	9q34.3	Köhler et al. (2013) [30]	rs10781500	Italy	DTC	1.23	3.5 × 10^− 5^
PLAU	10q22.2	Köhler et al. (2013) [30]	rs2633322	Italy	DTC	1.21	5.3 × 10^− 3^
SNX19	11q24.3-q25	Köhler et al. (2013) [30]	rs11823005	Italy	DTC	1.35	1.7 × 10^− 3^
GTSCR1	18q22.2	Köhler et al. (2013) [30]	rs9951245	Italy	DTC	1.20	9.8 × 10^− 4^
BATF	14q24.3	Figlioli et al. (2014) [31]	rs10136427	Italy	DTC	1.40	4.4 × 10^− 7^
DHX35	20q11.23-q12	Figlioli et al. (2014) [31]	rs7267944	Italy	DTC	1.39	2.1 × 10^− 8^
ARSB	5q14.1	Figlioli et al. (2014) [31]	rs13184587	Italy	DTC	1.28	8.5 × 10^− 6^
SPATA13	13q12.12	Figlioli et al. (2014) [31]	rs1220597	Italy	DTC	1.26	3.3 × 10^− 6^
GPD1L	3p22.3	Figlioli et al. (2014) [31]	rs1159444	Italian, Polish, Spanish	DTC	1.23	9.13 × 10^− 4^
TIPRL	1q24.2	Figlioli et al. (2014) [31]	rs2281016	Italian, Polish, Spanish	DTC	1.16	2.0 × 10^− 3^
DACH1	13q21.33	Figlioli et al. (2014) [31]	rs2245026	Italian, Polish, Spanish	DTC	1.17	2.0 × 10^− 3^
GALNTL4	11p15.4	Figlioli et al. (2015) [32]	rs7935113	Italian	DTC	1.36	7.4 × 10^− 7^
FOXA2	20p11.21	Figlioli et al. (2015) [32]	rs1203952	Italian	DTC	1.29	4.4 × 10^− 6^
CAMTA1	1p36.31-p36.23	Figlioli et al. (2015) [32]	rs10864251	Italian	DTC	1.17	1.40 × 10^− 3^
		Figlioli et al. (2015) [32]	rs4908581	Italian	DTC	1.22	4.61 × 10^− 5^
LOC728241	2	Figlioli et al. (2015) [32]	rs1400967	Italian	DTC	1.22	7.11 × 10^− 4^
C3orf63	3p14.3	Figlioli et al. (2015) [32]	rs11130536	Italian	DTC	1.24	3.27 × 10^− 4^
PDZRN3	3p13	Figlioli et al. (2015) [32]	rs3863973	Italian	DTC	1.22	5.95 × 10^− 4^
SYK	9q22.2	Figlioli et al. (2015) [32]	rs290212	Italian	DTC	1.23	6.84 × 10^− 5^
C14orf147	14q13.1	Figlioli et al. (2015) [32]	rs4624074	Italian	DTC	1.20	1.46 × 10^− 4^
WDR11-AS1	10q26.12	Mancikova et al. (2015) [33]	rs2997312	Southern European	NMTC	1.35	1.2 × 10^− 4^
		Mancikova et al. (2015) [33]	rs10788123	Southern European	NMTC	1.26	5.2 × 10^− 4^
		Mancikova et al. (2015) [33]	rs1254167	Southern Europea	NMTC	1.38	5.9 × 10^− 5^
HTR1B	6q14.1	Mancikova et al. (2015) [33]	rs4075570	Southern European	NMTC	0.82	2.0 × 10^− 4^
PCNXL2	1q42.2	Son et al. (2017) [29]	rs4649295	Korean	PTC	1.45	8.53 × 10^− 8^
VAV3	1p13.3	Son et al. (2017) [29]	rs4915076	Korean	PTC	1.34	7.09 × 10^− 8^
MSRB3	12q14.3	Son et al. (2017) [29]	rs11175834	Korean	PTC	1.36	4.86 × 10^− 7^
SEPT11	4q21.1	Son et al. (2017) [29]	rs1874564	Korean	PTC	1.31	5.87 × 10^− 7^
FHIT	3p14.2	Son et al. (2017) [29]	rs9858271	Korean	PTC	1.30	2.76 × 10^− 8^
INSR	19p13.2	Son et al. (2017) [29]	rs7248104	Korean	PTC	1.23	1.64 × 10^− 5^
SLC24A6 (SLC8B1)	12q24.13	Son et al. (2017) [29]	rs16934253	Korean	PTC	1.36	0.0216

^a^Non-Medullary Thyroid Cancer (NMTC), Papillary Thyroid Cancer (PTC), Differentiated Thyroid Cancer (DTC)

### Moderate and high penetrant mutations

#### FOXE1

The *FOXE1* (forkhead factor E1) gene is located at chromosome 9q22.33 and encodes for the *FOXE1* transcription factor (thyroid transcription factor 2, *TTF-2*), which regulates thyroglobulin and thyroperoxidase gene expression. In a genome-wide association study in a population of 192 and 37,196 thyroid cancer cases and controls, seven of the nine strongest association signals were in a similar linkage disequilibrium region as the *FOXE1* gene. Further replication results from 241 patients in combination with results from a GWAS showed the strongest association signal for allele A of *rs965513* with an OR of 1.75 in European populations from Iceland, Columbus, and Spain [35]. In a study by Landa et al. (2009), another single nucleotide polymorphism (SNP) (*rs1867277*) located in the 5’UTR region of *FOXE1* gene was positively associated with thyroid cancer in Spanish and Italian cohorts. The authors proposed this variant as a causal SNP in susceptibility to thyroid cancer through DNA binding assays and transfection studies. The variant was observed to cause cancer susceptibility through the recruitment of USF1/USF2 transcription factors [36].

Furthermore, the first study between *FOXE1* gene and susceptibility to FNMTC was reported in 2012. Nine exonic and promoter variants of *FOXE1* gene were studied in a population of 60 Portuguese FNMTC probands and 80 sporadic cases with matched controls (Table 2). As a result, *rs965513* and *rs1867277* were associated with increased risk of FNMTC. The authors also observed an association between FOXE1 polyalanine tract expansions and familial thyroid cancer risk (OR = 2.56) [37]. The same group identified a rare *FOXE1* variant (p.A248G) which co-segregated with thyroid cancer in one family and was also present in a case of sporadic NMTC. Further In vitro studies showed that this variant promoted cell migration and proliferation [38]. A large family-based study with 672 subjects belonging to 133 pedigrees with FNMTC cases genotyped twenty-three variants on 11 loci. Only three variants of 9q22.33 near *FOXE1* showed a positive association with FNMTC. *FOXE1* gene variant *rs1867277* had an OR of 3.17 under a recessive mode of inheritance. The other two variants also showed high penetrance under the recessive model (OR = 4.63 for rs10759944 and OR = 5.10 for rs965513) [39]. The *rs965513* variant has been previously associated with increased tumour size and extrathyroidal expansions in PTC patients [40]. In another two-step association study involving 1820 DTC cases and 2410 controls in Europe, two moderate penetrant *FOXE1* variants were identified, *rs7028661* with an OR of 1.64 and *rs7037324* with an OR of 1.54 [33]. He et al. showed that the *rs965513* variant and 4 other variants in close proximity regulate *FOXE1* and the PTC susceptibility candidate 2 (*PTCSC2*) gene transcriptional activity through regulatory enhancers [41]. *PTCSC2* is novel long non-coding RNA (lncRNA) gene with its transcripts downregulated in PTC tumours. Later, myosin-9 (MYH9) was identified as a PTCSC2 binding protein with the ability to inhibit the promoter shared by *FOXE1* and *PTCSC2* in both directions. Thus, PTC risk is potentially conferred by the interaction between a lncRNA (PTCSC2), MYH9, and FOXE1 [42].

**Table 2:** FOXE1 variants associated with hereditary thyroid cancer

FOXE1 variant	Annotation	Odds ratio*	***P***-value	Type of thyroid cancer	Population	Reference
rs965513	Intron variant	1.75	1.7 × 10^− 27^	NMTC	Iceland, Columbus, Spain	Gudmundsson et al. (2009) [35]
		2.81	< 0.0001	Sporadic NMTC	Portuguese	Tomaz et al. (2012) [37]
		2.30	0.0002	Familial NMTC		
		1.98 Additive Model	0.0045	Familial NMTC	NA	Bonora et al. (2014) [39]
		5.10 Recessive Model	0.00043	Familial NMTC		
		1.66	4.48 × 10^− 10^	PTC	Korean	Son et al. (2017) [29]
		1.587	4.2 × 10^− 4^	PTC	Japanese	Rogounovitch et al. (2015) [63]
		1.53	1.4 × 10^− 4^	PTC	Chinese	Wang et al. (2013) [67]
		1.65	4.8 × 10^− 12^	Radiation-related PTC	Belarus	Takahashi et al. (2010) [167]
		1.69	1.3 × 10^− 4^	Sporadic PTC	Japanese	Matsuse et al. (2011) [68]
rs7849497	5 prime UTR variant	2.14	0.0001	Familial + sporadic NMTC	Portuguese	Tomaz et al. (2012) [37]
rs1867278	5 prime UTR variant	1.7	0.0022	Familial + sporadic NMTC	Portuguese	Tomaz et al. (2012) [37]
rs1867279	5 prime UTR variant	2.17	< 0.0001	Familial + sporadic NMTC	Portuguese	Tomaz et al. (2012) [37]
rs1867280	5 prime UTR variant	1.62	0.0052	Familial + sporadic NMTC	Portuguese	Tomaz et al. (2012) [37]
rs1867277	5 prime UTR variant	1.70	0.0022	Familial + sporadic NMTC	Portuguese	Tomaz et al. (2012) [37]
		1.49	5.9 × 10^− 9^	PTC	Spanish and Italian	Landa et al. (2009) [36]
		3.17 Recessive Model	0.0013	Familial NMTC	NA	Bonora et al. (2014) [39]
rs3021523	Synonymous variant	2.04	0.0002	Familial + sporadic NMTC	Portuguese	Tomaz et al. (2012) [37]
rs10759944	Intron Variant	4.63 Recessive Model	0.00094	Familial NMTC	NA	Bonora et al. (2014) [39]
		2.06 Additive Model	0.0031	Familial NMTC	NA	Bonora et al. (2014) [39]
rs7037324	Non-coding transcript variant	1.54	1.2 × 10^− 17^	NMTC	Spain and Southern Europe	Mancikova et al. (2015) [33]
rs7028661	Intron variant	1.64	1.0 × 10^− 2^	NMTC	Spain and Southern Europe	Mancikova et al. (2015) [33]
		1.56	1.64 × 10^− 8^	PTC	Korean	Son et al. (2017) [29]
rs1588635	78 kb 5′ of FOXE1	1.57	1.30 × 10^− 8^	PTC	Korean	Son et al. (2017) [29]
rs10122541	NA	1.54	1.1 × 10^− 17^	NMTC	Spain and Southern Europe	Mancikova et al. (2015) [33]
rs3021526	Exon variant	1.85	0.0004	Familial + sporadic NMTC	Portuguese	Tomaz et al. (2012) [37]
PolyAla	PolyAla tract expansions (> 14 alanines)	2.49	< 0.0001	Familial + sporadic NMTC	Portuguese	Tomaz et al. (2012) [37]

*Odds ratios are for mode of inheritance unless mentioned otherwise

#### HABP2

The Hyaluronan-Binding Protein 2 *(HABP2)* gene is located on chromosome 10q25.3 and encodes a member of the peptidase S1 family of serine proteases [43]. Mutations in this gene are associated with non-medullary thyroid cancer and susceptibility to venous thromboembolism. Gara et al. (2015) performed whole-exome sequencing of peripheral blood in 7 affected members of a FNMTC kindred and unaffected spouses as controls, a germline variant (*G534E*; *rs7080536*) was identified in the *HABP2* gene. All affected family members were heterozygous for the variant in peripheral blood DNA. This mutation was found with an allele frequency of 2.2% in the ExAC database, and in 4.7% of 423 patients with sporadic thyroid cancer reported in Human Cancer Genome Atlas multiethnic database. Functional studies confirmed this loss of function variant’s pathogenicity and showed normal *HABP2* has tumour-suppressive functionality [43]. Zhou et al. [44], Sponziello et al. [45], and Tomsic et al. [46] later argued that the allele frequency of the *G534E* variant exceeds the filtering criterion used by Gara et al. (less than 1% in public databases) and the role of this variant in thyroid cancer requires more studies. A replication study by Zhang et al. with a cohort of 64 subjects from 29 kindred, identified *G534E* variant in 6 PTC patients from 4 independent kindreds. The prevalence rate of 13.8% was reported for this variant in the 29 kindreds, suggesting *HABP2* as a susceptibility gene for hereditary thyroid cancer [47]. However, NMTC risk conferred by *HABP2* G534E was not confirmed by an association study of over 2000 NMTC cases and over 5000 population controls from the British Isles. The frequency of *HABP2 G534* variant was 4.2% in cases and 4.6% in controls (OR = 0.74; *P* = 0.017) [48]. The data from various ethnic populations with large sample sizes suggest that this variant is unlikely to be a moderate or high penetrance gene in NMTC patients. Multiple other groups were unable to verify an association between the *G534E* variant and hereditary thyroid cancer [49–57]. Additionally, targeted sequencing of 516 PTC cases failed to identify the *G534E* variant. However, three other *HABP2* variants (*rs138864377, rs2286742, and rs3740530*) were identified that can potentially increase the risk of PTC. The *rs2286742* and *rs3740530* variants in *HABP2* had odds ratio of 9.644 and 3.989 in a recessive model, respectively (Table 3) [56]. However, no replication studies have been performed to identify the pathogenicity of these three *HABP2* novel variants in PTC.

**Table 3:** HABP2 variants associated with hereditary thyroid cancer

HABP2 variant	annotation	Odds ratio	***P***-value	Type of thyroid cancer	population	Reference
rs2286742	Intronic variant	9.644 Recessive model	0.026	PTC	NA	Shen et al. (2019) [56]
rs3740530	Synonymous variant	3.989 Recessive model	0.009	PTC	NA	Shen et al. (2019) [56]

### Low penetrance mutations

#### TITF1/ NKX2.1 and PTCSC3

*TITF1/NKX2.1* consists of two exons that encode thyroid-specific transcription factor-1 (TTF-1). A GWAS in the Icelandic population by Gudmundsson et al. identified an association between intergenic variant (*rs944289*) on chromosome 14q13.3 and risk for DTC (OR = 1.37) (Table 4) [35]. The closest gene to this variant is *NKX2.1* [58]. This association was also confirmed by a case-control study in 1085 Korean DTC cases and 8884 controls that yielded an OR of 1.23 for *rs944289* and an OR of 1.25 for the *rs34081947* variant [29]. Another case-control study from the northern Chinese Han population also found an association between the *rs944289* variant and PTC risk (OR = 1.23) [59]. On the contrary, a large family-based study of 672 subjects from 133 pedigree was not able to find any association between familial NMTC and *rs944289* [39]. Studies on the *rs944289* variant in different populations are summarized in Table 4. Ngan et al. performed targeted DNA sequencing for germline mutations in *TITF-1/NKX2.1* in 20 patients with multinodular goiter (MNG) and PTC, 284 with only PTC, and 349 controls. In 4 of 20 unrelated patients with MNG/PTC, a germline mutation (A339V) was identified in *NKX2.1/TITF-1*. Only two of these 4 patients had a positive family history of PTC and the mutation showed an autosomal dominant pattern of inheritance. The mutation was not found among 349 healthy control subjects or among the 284 PTC patients who had no history of MNG [60]. In another study, none of the 63 familial PTC cases had the A339V mutation [61].

**Table 4:** TITF1/NKX2.1 variant rs944289 associated with hereditary thyroid cancer

TITF1/NKX2.1 variant	Annotation	Odds ratio	***P***-value	Type of thyroid cancer	population	Reference
rs944289	NA	1.37	2.0 × 10^−9^	NMTC	Iceland, Columbus, Spain	Gudmundsson et al. (2009) [35]
		1.23	0.002	PTC	northern Chinese Han populations	Zhang et al. (2020) [59]
		1.24	0.0014	DTC	Korean	Son et al. (2017) [29]
		1.24	1.5 × 10^− 5^	NMTC	Spain and Southern Europe	Mancikova et al. (2015) [33]
		1.21	0.0121	Sporadic PTC	Japanese	Matsuse et al. (2011) [68]
		1.53	2.2 × 10^− 10^	PTC	Chinese	Wang et al. (2013) [67]
		1.23	0.003	PTC	Japanese	Rogounovitch et al. (2015) [63]
rs368187	exon variant	1.39	5.1 × 10^− 23^	NMTC	European ancestry	Gudmundsson et al. (2017) [28]
rs34081947	NA	1.27	1.2 × 10^− 7^	DTC	Korean	Son et al. (2017) [29]

Jendrzejewski et al. identified a non-coding RNA gene named papillary thyroid carcinoma susceptibility candidate 3 (*PTCSC3*) in a transcriptome gene expression analysis from 46 PTC tumour and unaffected thyroid tissue samples. Interestingly, *PTCSC3* is located 3.2 kb downstream of *rs944289* at 14q.13.3 and has lower expression in PTC thyroid tumours, suggesting a tumour-suppressor role. *PTCS3* expression is reduced by the T allele of the *rs944289* SNP which affects promoter activation. As a result, the risk allele of *rs944289* can potentially decrease *PTCSC3* promoter activation and thereby acts as a predisposition to PTC [62]^,^ [63].

#### SRGAP1

He et al. performed a genome-wide linkage analysis in 38 families with PTC and identified Slit-Robo GTPase-activating protein 1 (*SRGAP1*) as a candidate gene on chromosome 12q14.2. The SNPs, *rs781626187* **(**Q149H) and *rs797044990* **(**A275T) were two loss-of-function mutations in the Fes/CIP4 homology domain that segregated with PTC in one family each. Additionally, a missense variant (*rs114817817*) in the RhoGAP domain (R617C) also occurred in only one family [64]. The protein encoded by this gene is a GTPase activator and mutations in this gene can severely impair the ability to inactivate CDC42. CDC42 can mediate multiple signaling pathways, and plays a role in PTC tumourigenesis [65, 66]. To assess the frequency of the 4 missense variants in sporadic PTC cases and healthy controls, He et al. performed further targeted association studies on 2 large cohorts from Ohio and Poland which failed to confirm this association. In fact, *Q149H* and *A275T* were not found in 367 cases and 552 controls from Ohio or in the 432 cases and 424 controls from Poland. However, a SNP (*rs2168411*) located in intron 4 of *SRGAP1* showed an association with PTC in both Ohio and Poland cohorts with a combined OR of 1.21 (95% CI 1.08–1.35, *P* = .0008). The *rs114817817* variant was also identified in 4 of 742 sporadic cases of PTC in Ohio but in none of the 828 controls, which is suggestive of low penetrance. Future replication studies are required to confirm the candidacy of this variant [64].

#### NRG1

Previously, a SNP (*rs2439302*) on chromosome 8p12 was reported to be associated with PTC [27]. This association has been confirmed in multiple replication studies in Icelandic, Korean, Japanese, and Chinese populations (Table 5) [28, 63, 67]*.* The *rs2439302* variant has been confirmed as a PTC risk variant with odds ratios ranging from 1.2 to 1.4. This variant has also been correlated with multifocality and lymph node metastasis in PTC patients [40]. Another variant of NRG1 locus (SNP *rs2466076*) was found to have an OR of 1.32 among 3001 NMTC cases and 287,550 controls [65]. Both SNPs are located in the intronic regions of the neuregulin 1 (*NRG1*) gene. The *NRG1* gene encodes a membrane glycoprotein that mediates cell-cell signalling and plays a critical role in the growth and development of multiple organ systems.

**Table 5:** NRG1 variants associated with hereditary thyroid cancer

NRG1 variant	Annotation	Odds ratio	P-value	Type of thyroid cancer	population	Reference
rs2439302	Intron variant	1.36	2.0 × 10^− 9^	NMTC	Icelandic	Gudmundsson et al. (2012) [27]
		1.41	2.78 × 10^− 5^	PTC	Chinese	Wang et al. (2013) [67]
		1.27	0.003	PTC	Japanese	Rogounovitch et al. (2015) [63]
		1.46	4.0 × 10^− 5^	PTC	Kazakh	Mussazhanova et al. (2020) [168]
		1.59	2.45 × 10^− 5^	PTC	Ohio	Liyanarachchi et al. (2013) [70]
		1.23	9.29 × 10^− 4^	PTC	Poland	Liyanarachchi et al. (2013) [70]
rs2466076	Intron variant	1.32	1.5 × 10^− 17^	NMTC	Icelandic	Gudmundsson et al. (2017) [28]
rs6996585	Intron variant	1.43	9.0 × 10^− 12^	PTC	Korean	Son et al. (2017) [29]
rs12542743	Intron variant	1.39	1.01 × 10^− 10^	PTC	Korean	Son et al. (2017) [29]
rs2439304	Promoter variant	1.2	0.001	DTC	Europeans, Melanesians and Polynesians	Guibon et al. (2021) [74]

Additionally, NRG1 dysregulation is closely linked to *PI3K-AKT* and *MAPK* signalling pathways and has been demonstrated to be involved in tumourigenesis of both malignant and benign thyroid tumours [69, 70]. He et al. evaluated candidate functional variants of *NRG1*. The [G] risk allele (*rs2439302*) was associated with higher expression of the three tested isoforms in normal thyroid tissue. The authors proposed these isoforms as contributing factors to higher PTC risk through allele-specific enhancer-mediated transcriptional regulation of *NRG1* [71]. *NRG1* expression was also shown to be essential for PTC cell proliferation through protection from reactive oxygen species (ROS) damage by nuclear factor E2-related factor 2 (NRF2). Therefore, *NRG1* can also be useful as a potential therapeutic target for PTC patients [72, 73]. Guibon et al. performed fine-mapping of the 8p12 (*NRG1*) locus in Europeans, Melanesians and Polynesians populations and identified *rs2439304* associated with DTC (OR = 1.2). This variant had the highest posterior probability (PP) of causality in the three ethnic groups based on expression Quantitative Trait Locus (eQTL) data at this locus [74]. *NRG1* variants show stronger association in the Korean population compared to the European populations, suggesting a potential Korean-specific marker for DTC [29, 75].

#### DIRC3

*DIRC3* (Disrupted In Renal Carcinoma 3) is an RNA gene affiliated with the lncRNA class of RNAs. Several diseases have been associated with *DIRC3*, including renal cell, breast, and thyroid carcinoma. Multiple reports have demonstrated the prognostic significance of the *rs966423* variant of the *DIRC3* gene and its pathogenic effects in DTC cases. *DIRC3* was first identified in 2003 as a fusion transcript involved in familial renal carcinoma. Although the function of DIRC3 is still unknown, it is thought to have tumour suppressor activity [76]. In a GWAS with 561 Icelandic individuals with thyroid cancer cases and 40,013 controls, *DIRC3* variants were associated both with thyroid cancer risk and thyroid stimulating hormone levels. One variant that was significantly correlated with PTC was *rs966423* with an OR of 1.34 [27]. In the replication studies by Köhler et al. (2013) and Son et al. (2017) three other intronic variants in this gene were identified in DTC and PTC cases with low penetrance (Table 6) [29, 30]. However, Mankickova et al. were unable to establish an association between *rs966423* and thyroid cancer in a European population, suggesting inter-population heterogeneity in thyroid cancer susceptibility [33]. Patients homozygous for the T allele of *rs966423* have a 6.4% higher mortality risk compared to CC/CT carriers (*P* = 0.017) [77]. Additionally, CT genotype carriers were associated with extrathyroidal extension and more advanced T stage [78]. On the contrary, a recent study in 1466 DTC patients reported no association between any genotype at the *rs966423* SNP and overall mortality and response to therapy [79]. A recent GWAS analysis also identified five novel variants including *rs11693806* as a non-coding variant located close to DIRC3 in a large sample of 2637 European ancestry cases and 134,811 European ancestry controls [28]. Another study by Guibon et al. identified *rs16857609* as a novel variant located near DIRC3. This SNP was associated with DTC in the European population (OR = 1.4, *p* = 1.9 × 10^− 10^) [74]. Future studies should replicate the findings of the known DIRC3 variants and confirm their association with PTC pathogenesis.

**Table 6:** DIRC3 variants associated with hereditary thyroid cancer

DIRC3 variant	Annotation	Odds ratio	P-value	Type of thyroid cancer	Population	Reference
rs6759952	Intron variant	1.3	7.3 × 10^−8^	DTC	Italian	Köhler et al. (2013) [30]
		1.21	0.0164	DTC	Korean	Son et al. (2017) [29]
rs11693806	Non-coding transcript variant	1.43	1.5 × 10^− 24^	NMTC	European	Gudmundsson et al. (2017) [28]
rs966423	Intron variant	1.34	1.3 × 10^− 9^	NMTC	Iceland, Spain, United States, Netherlands	Gudmundsson et al. (2012) [27]
		1.27	0.0067	PTC	Korean	Son et al. (2017) [29]
		1.31	0.001	PTC	Chinese	Wang et al. (2013) [67]
		1.28	2.12 × 10^− 2^	PTC	Ohio	Liyanarachchi et al. (2013) [70]
		1.14	2.94 × 10^− 2^	PTC	Polish	Liyanarachchi et al. (2013) [70]
		1.18	0.07	PTC	Kazakh	Mussazhanova et al. (2021) [168]
rs12990503	Intron variant	1.38	2.58 × 10^− 10^	PTC	Korean	Son et al. (2017) [29]
rs16857609	NA	1.42	3.7 × 10^− 10^	DTC	European	Guibon et al. (2021) [74]

### Polygenic risk score

As reviewed in the previous section and summarized in Table 1, the genome-wide association studies identified many low penetrant risk alleles for thyroid cancer. Single genetic variants with such low-risk alleles do not explain the clustering of thyroid cancer in families. Consequently, polygenic risk scores (PRS) have been developed to consider panels of SNPs to calculate their additive risk for thyroid cancer. The integration of PRS with family history can potentially improve identifying people at risk for developing thyroid cancer in various populations. A recent study investigated the combined genetic effects of 10 well-established SNPs (*rs12129938, rs11693806, rs6793295, rs73227498, rs2466076, rs1588635, rs7902587, rs368187, rs116909374, and rs2289261*) associated with PTC by evaluating their PRS with data from previous GWAS from United States, Iceland, and the United Kingdom. Their results indicate a 6.9-fold greater risk for thyroid cancer for patients in the top decile of the ten common SNPs polygenic risk scores compared to the bottom decile [80]. Similarly, Hoang et al. investigated the value of PRS for thyroid cancer in a Korean population. In this study, a family history of thyroid cancer (OR = 2.96), obesity (OR = 1.72), weighted (OR = 1.56), and unweighted PRS (OR = 1.46) were associated with thyroid cancer susceptibility [81]. The PRS of 12 thyroid cancer-associated SNPs (*rs11693806, rs2466076, rs1588635, rs368187, rs116909374, rs12129938, rs6793295, rs73227498, rs7902587, rs2289261, and rs56062135*) was investigated in 2370 childhood cancer survivors with an European ancestry. Similar to previous findings, the hazard ratio for developing secondary thyroid cancer by one standard deviation increase in the PRS was 1.57 (95% CI = 1.25–1.83; *P* <  0.001) [82]. Likewise, in a phenome-wide association study of 472 thyroid cancer patients with European ancestry, a PRS of 9 SNPs exhibited a strong association with thyroid cancer (OR = 3.2) when the top PRS quartile was compared to the bottom quartile [83]. In a study by Wang et al., Individuals with African ancestry who were in the top PRS quintile of 5 SNPs had a 30% greater chance of thyroid cancer (OR = 1.3) than those in the lowest quintile [84]. Additionally, in a study with 495 thyroid cancer patients and 56,439 controls by Song et al., the PRS of 6 SNPs (*rs6759952, rs13059137, rs7834206, rs72616195, rs1369535, rs11175834*) increased thyroid cancer risk by a factor of 3.9 when comparing high PRS tertile with low PRS tertile [85]. Given the presented findings, PRS has the potential to identify individuals at a higher risk of thyroid cancer. However, studies with larger sample sizes and more inclusive PRS with wide varieties of SNPs are required for determining the optimal PRS model for thyroid cancer.

## Rare germline mutations in families with Non-syndromic familial non-medullary thyroid cancer

Non-syndromic familial non-medullary thyroid cancer (NSFNMTC) accounts for 95% of FNMTC cases. The genetic risk factors of non-syndromic FNMTC are poorly understood compared to familial NMTC associated with hereditary syndromes (syndromic NMTC). In addition to *FOXE1, HABP2, NRG1, SRGAP1, DIRC3, TITF1/ NKX2.1* and *PTCSC3,* multiple other genes and chromosomal loci have been linked to families affected by non-syndromic FNMTC in linkage studies and/or whole-exome/whole-genome sequencing studies. The identified mutations are present in only a subset of FNMTC kindreds and require further validation studies. Table 7 summarizes multiple studies that investigated the genetic component of FNMTC in families with NSFNMTC.

**Table 7:** Genes and chromosomal loci linked to non-syndromic familial non-medullary thyroid cancer

Gene	Chromosome	Study details	Reference
MAP2K5	15q23	34 families with two first-degree relatives with PTC (no syndromic FNMTC). Whole exome and target gene sequencing for candidate variants.	Ye et al. (2019) [86]
PLCB1	20p12.3	Genome wide linkage analysis and next generation sequencing performed in a family with MNG that was likely to progress to PTC as seen in some family members. An intronic PLCB1 InDel was found in all affected members.	Bakhsh et al. (2018) [87]
BROX	1q41	Whole-exome sequencing of PTC patients from five families. Two BROX variants were observed in two of the families.	Pasquali et al. (2021) [88]
POT1	7q31.33	Whole exome sequencing of five affected family members with melanoma and thyroid cancer revealed a new mutation in POT1. POT1 is involved with the telomere shelterin complex that controls telomere protection.	Wilson et al. (2017) [89]
		A low frequency variant in POT1 was found in childhood cancer survivors that developed thyroid cancer.	Richard et al. (2020) [90]
		A POT1 variant causes telomere dysfunction in a family affected only by FNMTC.	Srivastava et al. (2020) [91]
ATM	11q22.3	Whole-genome sequencing and genome-wide linkage analysis in 17 FNMTC families. ATM variant was identified in two families.	Wang et al. (2019) [92]
CHEK2	22q12.1	Whole-genome sequencing and genome-wide linkage analysis in 17 FNMTC families. A CHEK2 (breast and prostate cancer susceptibility gene) variant was identified in one family.	Wang et al. (2019) [92]
NOP53	19q13.33	Exome sequencing in a family with five cases of NSFNMTC and 44 additional families with FNMTC showed a low-penetrance germline variant of NOP53 with increased levels in tumour samples of the affected cases.	Orois et al. (2019) [93]
NDUFA13/ GRIM-19	19p13.11	A germline mutation was found in a patient with Hurthle cell PTC. No Grim-19 mutations were observed in familial Hurthle cell tumours. GRIM-19 is involved in mitochondrial metabolism.	Máximo et al. (2005) [94]
TIMM44	19p13.2	Screening of 14 candidate genes in the linkage region of affected TCO members from 8 FNMTC families. TIMM44 is a mitochondrial inner membrane translocase.	Bonora et al. (2006) [95]
SRRM2	16p13.3	Whole exome sequencing in a PTC family with six affected first- or second-degree relatives detected a germline variant in SRRM2. This gene is involved in RNA splicing, with aberrant alternative splicing in affected individuals.	Tomsic et al. (2015) [96]
ANXA3	4q21.21	Whole exome sequencing in three Brazilian families with familial PTC yielded seven new genes with implication in hereditary PTC.	Sarquis et al. (2020) [97]
NTN4	12q22		
SERPINA1	14q32.13		
FKBP10	17q21.2		
PLEKHG5	1p36.31		
P2RX5	17p13.2		
SAPCD1	6p21.33		
Unknown	8q24 (PTCSC1)	Genome wide linkage analysis in a large family with PTC and melanoma.	He et al. (2009) [98]
Unknown	4q32	Linkage analysis and targeted deep sequencing identified an ultra-rare mutation (SNP) in chromosome 4q32 in a large pedigree affected by FNMTC.	He et al. (2013) [99]
Unknown	6q22	Linkage analysis in 38 FNMTC families revealed, 6q22 (Maximum LOD of 3.3) displayed linkage.	Suh et al. (2009) [100]
Unknown	1q21 (fPTC,PRN)	Linkage analysis in a large three-generation familial PTC kindred, maximum LOD of + 3.58.	Malchoff et al. 2000) [101]
		Linkage analysis in forty-nine affected cases with FNMTC, maximum LOD of + 3.04.	Suh et al. (2009) [100]
Unknown	14q32 (MNG1)	Linkage analysis in a Canadian family with 18 cases of MNG (2 of which were also diagnosed with PTC). Maximum LOD of 3.8.	Bignell et al. (1997) [102]
Unknown	2q21 (NMTC1)	Linkage analysis in a large Tasmanian pedigree with PTC. Multipoint heterogeneity LOD of 3.07.	Mckay et al. (2001) [103]
		Linkage analysis in 10 FNMTC families. Linkage evident at both TCO and NMTC (LOD = 1.56 and 2.85, respectively)	Mckay et al. (2004) [104]
		Loss of heterozygosity (LOH) was analyzed at 2q21 and 19p13.2 in 9 FNMTC families. Two of the fourteen tumours displayed LOH at 2q21 (14%).	Prazeres et al. (2008) [105]
Unknown	8p23.1-p22 (FTEN)	Linkage analysis in a Portuguese family affected by PTC and benign thyroid lesions detected a linkage with 8p23.1-p22, Maximum haplotype-based LOD of 4.41.	Cavaco et al. (2008) [106]
Unknown	19q13.2 (TCO)	Linkage analysis in a French pedigree affected by PTC and MNG lead to mapping chromosome 19p13.2 to TCO (thyroid tumours with cell oxyphilia). Maximum LOD of 3.01.	Canzian et al. (1998) [107]
		Linkage analysis in one family with PTC and MNG. Maximum LOD of 1.54.	Bevan et al. (2001) [108]
		Linkage analysis in ten families affected by PTC and MNG. Maximum LOD of 1.56.	McKay et al. (2004) [104]
		Loss of heterozygosity (LOH) was analyzed at 19p13.2 in 9 FNMTC families. Eight of the fourteen tumours displayed LOH at 19p13.2 (57%).	Prazeres et al. (2008) [105]

## Syndromic familial non-medullary thyroid cancer

Hereditary syndromes (syndromic FNMTC) with various clinical features may be associated with approximately 5% of familial non-medullary thyroid cancer cases (Table 8). In addition to the implicated syndrome or disease symptoms, patients with syndromic FNMTC may develop cancers of non-thyroidal origin as well.. In a recent study, Zhou et al. checked twenty-five candidate NMTC susceptibility genes against six genetic resources including ClinGen, NCCN guidelines, OMIM, Genetics Home Reference, GeneCards, and Gene-NCBI. These susceptibility genes were assessed based on gene-disease association from previous studies. Subsequently, 12 genes (*APC, DICER1, FOXE1, HABP2, NKX2–1, PRKAR1A, PTEN, SDHB, SDHD, SRGAP1, CHEK2, and SEC23B*) were verified as NMTC susceptibility genes. Seventy-nine diseases were associated with these 12 susceptibility loci, some of which are causative genetic components of syndromic FNMTC, while others have been implicated in non-syndromic FNMTC [109]. The predominant syndromes that may lead to the development of syndromic NMTC are familial adenomatous polyposis (FAP), Cowden’s disease, Carney’s complex type 1, Werner’s syndrome, DICER1 syndrome, Li-Fraumeni syndrome, PTEN hamartoma tumour syndrome, Peutz-Jeghers syndrome, Bannayan-Riley-Ruvalcaba syndrome, Ataxia-telangiectasia, and Pendred syndrome. Syndromic FNMTC susceptibility genes and their highly penetrant mutations could be of great value for screening at-risk individuals, thereby making early diagnosis and selecting appropriate treatment possible. It is important for clinicians to recognize the phenotypes of these syndromes so that genetic counselling can be initiated to enable surveillance for associated malignancies and genetic testing of family members. Additionally, more frequent screening is warranted for first-degree family members of patients affected by syndromic FNMTC.

**Table 8:** Hereditary syndromes associated with thyroid cancers of follicular cell origin

Name	Mode of Inheritance	Responsible gene	Chromosome	Thyroid cancer histological subtype	Phenotypes other than thyroid cancer
FAP and Gardner’s syndrome	Autosomal dominant	APC	5q21	PTC with cribriform pattern	Colorectal carcinoma, ampullary carcinoma, hepatoblastoma, medulloblastoma
Cowden Syndrome	Autosomal dominant	PTEN, SDHB-D, PIK3CA, AKT1,KLLN,SEC23B	10q22–23 1p36.13 3q26.32 14q32.33 10q23.31 20p11.23	PTC (classical and follicular variants) FTC	Multiple hamartomas, follicular thyroid carcinoma, benign thyroid nodules, breast cancer, endometrial cancer
Werner syndrome	Autosomal recessive	WRN	8p11–21	PTC, FTC, ATC (anaplastic thyroid carcinoma)	Premature aging, scleroderma-like skin changes, cataracts, subcutaneous calcifications, muscular atrophy, diabetes
Carney complex	Autosomal dominant	PRKAR1	17q22–24	PTC, FTC	Spotty skin pigmentation, cardiac myxomas, endocrine tumours
DICER1 syndrome	Autosomal dominant	DICER1	14q32.13	PTC, DTC	Endocrine tumours (thyroid, parathyroid, pituitary, pineal gland, endocrine pancreas, paragangliomas, medullary, adrenocortical, ovarian, and testicular tumours
Pendred syndrome	Autosomal recessive	SLC26A4, FOXI1, KCNJ10	7q21–34	PTC, FTC, ATC	Sensorineural deafness/hearing impairment, goiter, and an abnormal organification of iodide with or without hypothyroidism
Ataxia-telangiectasia	autosomal recessive	ATM	11q22–23	PTC	Cerebellar degeneration, telangiectasia, immunodeficiency, recurrent sinopulmonary infections, radiation sensitivity, premature aging, lymphoid cancer, poor growth, gonadal atrophy, insulin resistant diabetes
Bannayan-Riley- Ruvalcaba syndrome	autosomal dominant	PTEN	10q23.3	PTC, FTC	Macrocephaly, hamartomatous tissue overgrowth, lipomas, pigmented macules on the penis, developmental delay, large birth weight, joint hyperextensibility, endometrial cancer, renal cell carcinoma, Lhermitte–Duclos disease
Peutz-Jeghers syndrome	Autosomal dominant	STK11		PTC, DTC	Gastrointestinal (GI) polyposis, mucocutaneous pigmented macules, breast cancer, uterine cancer, cervical cancer, lung cancer, ovarian cancer, testicular cancers
PTEN hamartoma tumour syndrome	autosomal dominant	PTEN	10q23.31	FTC, PTC, fvPTC, MNG	Breast cancer, Endometrial cancer, FTC, Gastrointestinal hamartomas, Lhermitte-Duclos disease, Macrocephaly, Macular pigmentation of the glans penis, Multiple mucocutaneous lesions, Autism spectrum disorder, Colon cancer, Esophageal glycogenic acanthosis, Lipomas, Mental retardation, Renal cell carcinoma, Testicular lipomatosis, PTC, fvPTC, thyroid adenoma, MNG
Li-Fraumeni syndrome	Autosomal dominant	TP53	17p13.1	cPTC, FVPTC	Adrenocortical carcinomas, breast cancer, central nervous system tumours, osteosarcomas, soft-tissue sarcomas, leukemia, lymphoma, gastrointestinal cancers, cancers of head and neck, kidney, larynx, lung, skin, ovary, pancreas, prostate, and testis

### Familial adenomatous polyposis (FAP) and Gardner’s syndrome

Familial adenomatous polyposis (FAP) is an autosomal dominant disease caused by loss-of-function mutations of the *APC* tumour suppressor gene located on chromosome 5q21. The classic type of FAP is characterized by the development of multiple benign polyps lining the mucosa of the gastrointestinal tract, particularly the colon. Untreated polyps can become malignant with an early age of onset. Papillary thyroid carcinomas are seen in some families affected by FAP [110, 111]. In fact, patients with FAP have a 160-fold greater risk of PTC compared to the general population. The prevalence of thyroid cancer among patients with FAP is 2.6%. These thyroid cancers have a unique cribriform pattern on histologic examination and occur more commonly at a young age (< 30 years) in women (95%) [112, 113].

More than 60% of *APC* pathogenic mutations have been identified in the mutation cluster region between codons 1284 and 1580 [114, 115]. Most female patients with FAP and PTC also have a *RET* somatic mutation in addition to *APC* germline mutations in their tumours [116]. The APC gene encodes a multidomain protein that plays a significant role in tumour suppression by negatively regulating the WNT signalling pathway. Loss of *APC* function results in inappropriate activation of this pathway which results in cancer progression [117].

### Werner’s syndrome

Werner’s syndrome is an autosomal recessive disease characterized by premature aging, scleroderma-like skin changes, cataracts, subcutaneous calcifications, muscular atrophy, diabetes, and a high incidence of neoplasms, including thyroid neoplasms. Werner’s syndrome has been linked to mutations of the WRN gene on chromosome 8p11–21. This gene encodes a member of the RecQ subfamily of DNA helicase proteins that is important in maintaining genome stability by regulating DNA repair, replication, transcription, and telomere maintenance [118]. Thyroid cancer was observed in 16% of 189 patients with Werner syndrome in a Japanese case series. Follicular thyroid cancer was more common, followed by papillary and anaplastic thyroid cancers among these patients [119].

### Carney complex

Carney complex (CNC) is an autosomal dominant disease caused by mutations in the *PRKAR1* tumour suppressor gene mapped to chromosome 17q22–24 [120]. A loss of function mutation in *PRKAR1A* can lead to increased PKA signalling [121]. Additionally, this gene can fuse to the RET proto-oncogene by gene rearrangement and forming a thyroid tumour-specific chimeric oncogene known as PTC2. A loss of function mutation in *PARKARI* causes increased PKA signalling, leading to AMP-activated kinase (AMPK) activation through LKB1 kinase and increasing mTOR signalling [122]. As a result, patients may present with acromegaly, spotty skin pigmentation, an increased risk of cardiac and mucocutaneous myxomas, and a variety of tumours involving endocrine organs.

Additionally, about 60% of patients affected by CNC will develop thyroid tumours that range from follicular hyperplasia to multiple types of thyroid cancer, with follicular adenoma as the most common finding [7]. In a study by Stratakis et al. the prevalence of thyroid nodules and cancers in a series of 338 Carney’s complex patients was 5%, including follicular adenomas, PTC, follicular variant PTC (FvPTC), and FTC [123]. Patients affected by CNC should undergo surveillance using frequent ultrasound and biopsies to increase the likelihood of treatment success.

### DICER1 syndrome

DICER1 syndrome, also known as pleuropulmonary blastoma syndrome and dysplasia syndrome, is an autosomal dominant genetic disorder that predisposes individuals to various conditions, including benign and malignant tumours of different origins. Germline mutations of the *DICER1* gene located on 14q32.13 are detected in endocrine tumours (thyroid, parathyroid, pituitary, pineal gland, endocrine pancreas, paragangliomas, medullary, adrenocortical, ovarian, and testicular tumours).

The *DICER1* gene is a member of the ribonuclease III (RNaseIII) family involved in the generation of microRNAs (miRNAs) and modulates gene expression by interfering with mRNA function. *DICER1* germline loss-of-function mutations disrupt the correct timing and expression of miRNA production necessary for normal thyroid differentiation and function [124, 125]. Khan et al. investigated the risk of thyroid cancer in 145 individuals with *DICER1* germline mutations and 135 family controls from 48 families. This group reported a 16-fold increased risk of thyroid cancer, with all the cases harbouring germline and somatic pathogenic *DICER1* mutations [126].

Thyroid abnormalities are common in DICER1 syndrome with multinodular goiter seen frequently in many families with a germline *DICER1* mutation. Thus, familial MNG is highly suggestive of DICER1 syndrome. In contrast, differentiated thyroid carcinoma (DTC) was infrequently seen in pedigrees with germline DICER1 mutation. However, multiple differentiated thyroid carcinomas have been found in three children with a history of prior chemotherapy and radiation exposure for the treatment of pleuropulmonary blastoma (PPB). As a result, there has been considerable speculation on a possible link between chemotherapeutic agents and an increased risk of differentiated thyroid cancer due to somatic DICER1 mutations [127]. More recently, a family study reported differentiated thyroid cancer and MNG in six individuals from a family with DICER1 pathogenic mutations and no history of chemotherapy [128].

### PTEN hamartoma tumour syndromes

PTEN hamartoma tumour syndrome (PHTS) consists of a group of disorders caused by germline mutations in the phosphatase and tensin homolog (*PTEN*) gene located at 10q23.31. They include Cowden syndrome, Bannayan-Riley-Ruvalcaba syndrome (BRRS), PTEN-related Proteus syndrome, Proteus-like syndrome, and adult Lhermitte-Duclos disease (LDD) [129, 130]. Approximately 6 to 38% of PHTS patients develop thyroid cancer with a median age of diagnosis of 31–37 years, indicating a risk 51 to 72 times higher than those without PHTS [130]. Therefore, the presence of a PTEN mutation justifies surveillance with annual neck palpation and ultrasound imaging starting at age 10 [131, 132].

PTEN is a phosphatase that counteracts the phosphatidylinositol 3-kinase (PI3K)/AKT signalling pathways. The tumour-suppressor activity of *PTEN* is thought to be associated with lipid dephosphorylation at the plasma membrane. An inactivated *PTEN* gene may increase PIP3 levels leading to AKT activation and mTOR signalling which in turn upregulates cell proliferation and survival while decreasing apoptosis [133, 134].

Patients with Cowden syndrome may suffer from breast, endometrium, colon, thyroid, and kidney tumours in addition to NMTC due to *PTEN* mutations. At least two-thirds of patients with this syndrome are affected by thyroid disease, often before the age of 20. In addition, approximately 10% of patients with Cowden syndrome will develop thyroid cancer (FTC or PTC) in their lifetime [7], [135]. In a study of 664 patients with Cowden syndrome (CS) or Cowden-like syndrome (CLS), 55.1% of the thyroid cancer cases were of classical papillary subtype. In this cohort, 5.4% of the CS and CLS patients had *PTEN* germline mutations. About 4% of the patients that did not harbor *PTEN* mutations tested positive for *SDHB-D* mutations and 2.3% tested positive for *KLLN* promoter methylation [136]. The SDHB-D genes located on chromosome 1p36.13 encodes succinate dehydrogenase (SDH). Its germline variants can result in the upregulation of the AKT and MAPK pathways, similar to *PTEN* mutations that can drive tumour formation [137]. *KLLN* is a tumour suppressor gene located upstream of PTEN. *KLLN* promoter methylation downregulates its transcription and disrupts p53-mediated activation of *KLLN* [138]. In another study, targeted sequencing of 91 CS and CLS probands without *PTEN, SDHB-D and KLLN* mutations, revealed PIK3CA germline mutations in 8.8% and AKT1 germline mutations in 2.2% of cases [139]. *PIK3CA* is a gene located on chromosome 3q26.32 and encodes p110a, the catalytic subunit of PI3K, which adds a phosphate to phosphatidylinositol-4,5-biphosphate (PIP2) to form phosphatidylinositol-3,4,5-triphosphate (PIP3) at the cellular membrane. PIP3 recruits AKT1 to the cell membrane. Subsequently, activated AKT phosphorylates downstream protein effectors, including the mammalian target of rapamycin (mTOR), which has an established role in human cancers [140].

Whole exome sequencing of a CS proband with the other family members affected with thyroid cancer across 4 generations was performed in 2015. Although all individuals tested negative for *PTEN, SDHB-D, KLLN* hypermethylation*, PIK3CA and AKT1*, several novel candidate genes were identified. All family members with CS shared 3 genes with heterozygous missense variants, *C16orf72* (c.253 T > C,p.Ser85Pro), *PTPN2* (c.1204G > A,p.Ala402Thr) and *SEC23B* (c.1781 T > G, p.Val594Gly). All 3 genes were sequenced in 96 unrelated CS probands with thyroid cancer, and germline heterozygous *SEC23B* variant was detected in 3 probands (3.1%). *SEC23B* encodes Sec23 Homolog B, a component of coat protein complex II (COPII) responsible for transporting proteins from the endoplasmic reticulum (ER) to the Golgi apparatus [141].

Bannayan-Rubalcaba-Riley syndrome (BRRS) is an overgrowth disorder with germline *PTEN* tumour-suppressor gene involvement in 60% of cases. BRRS involves macrocephaly, pigmented maculae of the glans penis, and benign mesodermal hamartomas. About 30% of BRRS patients may have diseases of thyroid origin, including, NMTC, thyroid adenoma, MNG, and Hashimoto’s disease [136, 142].

### Rare syndromes associated with NMTC

Studies involving patients with syndromic FNMTC have the power to add to the list of possible thyroid cancer susceptibility loci and help the identification of key players in thyroid tumorigenesis. Case reports and familial studies have identified multiple rare syndromes associated with a risk for NMTC. Li-Fraumeni syndrome caused by mutations in *TP53* gene and presents with a high risk of cancers with bone, breast, adrenal gland, and nervous system origins, with a lifetime cancer risk of > 70% for men and > 90% for women [143]. Formiga et al. established the presence of thyroid cancer in 193 Li-Fraumeni Syndrome (LFS) patients. 101 Out of 193 LFS cases, 101 were carriers of the Brazilian *TP53 p.R337H* mutation with 10.9% of cases exhibiting papillary thyroid carcinoma tumours [144].

Pendred syndrome is an autosomal recessive disorder characterized by bilateral sensorineural deafness and goitre caused by mutations in the *SLC26A4* (PDS) gene (7q12–34) [145]. The protein product of the *SLC26A4* gene is pendrin, a surface anion channel found on the apical membrane of thyroid follicular cells. A loss of function mutation in *SLC26A4* may disrupt iodine transport and result in goitre and hypothyroidism [146]. Additionally, follicular thyroid cancer, Hürthle cell adenoma, MNG, and fvPTC have been observed in families affected by Pendred syndrome [147–150]. Untreated congenital hypothyroidism, chronic stimulation by thyroid-stimulating hormone, and additional genetic alterations may also be involved in the formation of thyroid cancer in pendred patients [145, 149, 151].

Ataxia-telangiectasia syndrome is an autosomal recessive disorder caused by mutations in the (Ataxia Telangiectasia, Mutated) *ATM* gene on 11q22–23. The *ATM* gene encodes a member of the phosphatidylinositol 3-kinase family and plays a role in cellular responses to DNA breaks and oxidative stress. Patients with Ataxia-telangiectasia may present with cerebral ataxia, immunodeficiency, telangiectasia, radiation sensitivity, thymic atrophy, and various malignancies, particularly those with lymphoid origin [152]. Furthermore, mutations in the *ATM* gene have been implicated in PTC and fvPTC [153–157]. A Danish population-based study of 10,324 individuals identified an association between heterozygosity at ATM Ser707Pro and thyroid/endocrine cancer (HR = 10) [158]. Additionally, using whole-genome sequencing and genome-wide linkage analysis, Wang et al. identified *ATM* variants in 2 of 17 families affected by FNMTC [92].

Peutz-Jeghers syndrome (PJS) is an autosomal dominant disorder characterized by hamartomatous polyps, mucocutaneous hyperpigmentation, and a 4-fold increase in cancer risk compared to the general population [159]. Mutations in the *STK11* (serine/threonine-protein kinase 11 alias LKB1) gene (19p13.3) have been implicated as a causative agent for PJS. The protein product of the *STK11* gene is a serine-threonine kinase involved in second messenger signal transduction and AMPK inhibition [160]. Additionally, PJS has been associated with multiple cases of thyroid cancer of PTC, FTC, tall cell variant PTC, and fvPTC subtypes [159, 161, 162]. Papillary Renal Neoplasia (PRN) [101] and McCune–Albright syndrome [163] are two other rare disorders associated with risk of thyroid cancer. Nevertheless, only a few families have been affected by both thyroid cancer and the mentioned syndromes.

## Conclusion

Non-medullary thyroid cancer originates from follicular cells of the thyroid gland and accounts for the majority of thyroid cancers. The genetic component of NMTC tumourigenesis is strong but poorly understood, especially for familial NMTC. This review aimed to summarize the current understanding of genetic predisposition to NMTC by looking at genetic variants implicated in familial and sporadic NMTC. Increasing evidence suggests that mutations in the *FOXE1* gene have moderate to high penetrance. On the other hand, there is a lack of strong evidence for the role of *HABP2* mutations. Thus, further research is needed to clarify its role as a susceptibility gene in NMTC. Most of the mutations in *TITF1/ NKX2.1, PTCSC3, SRGAP1*, *NRG1, DIRC3* genes are low penetrant mutations. Although each low penetrant mutation does not seem to have clinical significance alone, a combination of these mutations could have clinical importance regarding hereditary NMTC.

Different germlines variants are only observed in small groups of FNMTC patients and may not be present in all affected family members within a kindred. Likewise, due to the lack of interventional screening programs, there are no genetic tests available to identify individuals at risk of FNMTC. As a result, the National Comprehensive Cancer Network (NCCN), the American Thyroid Association (ATA), and the European Society for Medical Oncology (ESMO) provide no recommendations on using genetic testing for screening at-risk family members of FNMTC patients [164–166]. We also do not think that we have enough evidences supporting the application of genetic screening for certain genes among patients with FNMTC, unless patients medical history and family history suggest a syndromic NMTC that should be tested for the related gene(s).

Further multi-center studies with larger cohorts and stricter inclusion criteria using targeted sequencing or whole exome/genome sequencing are needed to better understand the clustering pattern seen in the families with NMTC. Identification of NMTC susceptibility genes could potentially result in determining targeted treatment options for NMTC patients. Likewise, alternative hereditary mechanisms such as epigenetic modifications may also be involved in the pathogenesis of FNMTC and requires further research. Additionally, identifying new NMTC-associated genetic loci and research on the known implicated variants can improve our understanding of NMTC tumorigenesis in general, which could eventually result in earlier diagnosis and more effective treatment options for sporadic NMTC.

## Data Availability

Not applicable.

## Abbreviations

NMTC: Non-medullary thyroid cancer
DTC: Differentiated thyroid cancer
PTC: Papillary thyroid cancer
FNMTC: Familial non-medullary thyroid cancer
GWAS: Genome wide association studies
OR: Odd Ratio
*FOXE1*: Forkhead box E1
SNP: Single nucleotide polymorphism
TTF-1: Thyroid-specific transcription factor-1
NKX2.1: NK2 homeobox 1
lncRNA: Long non-coding RNA
MNG: Multinodular goiter
SRGAP1: Slit-Robo GTPase-activating protein 1
*HABP2*: Hyaluronan-Binding Protein 2
PTCSC2: PTC susceptibility candidate 2
PTCSC3: PTC susceptibility candidate 3
PRS: Polygenic risk score
AMPK: AMP-activated kinase
DIRC3: Disrupted In Renal Carcinoma 3
*NRG1*: Neuregulin 1
PP: Posterior probability of causality
MYH9: Myosin-9
FAP: Familial adenomatous polyposis
FTC: Follicular thyroid cancer
ATC: Anaplastic thyroid cancer
CS: Cowden syndrome
CLS: Cowden-like syndrome
CNC: Carney complex
SDH: Succinate dehydrogenase
PIP2: Phosphatidylinositol-4,5-biphosphate
PIP3: Phosphatidylinositol-3,4,5-triphosphate
PPB: Pleuropulmonary blastoma
PI3K: Phosphatidylinositol 3-kinase
mTOR: Mammalian target of rapamycin
ROS: Reactive oxygen species
eQTL: expression Quantitative Trait Locus
PHTS: PTEN hamartoma tumour syndrome
BRRS: Bannayan-Riley-Ruvalcaba syndrome
LDD: Lhermitte-Duclos disease
COPII: Coat protein complex II
ER: Endoplasmic reticulum
RNaseIII: Ribonuclease III
miRNAs: MicroRNAs
LFS: Li-Fraumeni Syndrome
